# The Mutational and Microenvironmental Landscape of Cutaneous Squamous Cell Carcinoma: A Review

**DOI:** 10.3390/cancers16162904

**Published:** 2024-08-21

**Authors:** Tara M. Hosseini, Soo J. Park, Theresa Guo

**Affiliations:** 1 Gleiberman Head and Neck Cancer Center, Moores Cancer Center, University of California San Diego, La Jolla, CA 92093, USA; 2 Division of Hematology-Oncology, Department of Medicine, University of California San Diego, La Jolla, CA 92093, USA; 3 Department of Otolaryngology-Head & Neck Surgery, University of California San Diego, La Jolla, CA 92093, USA

**Keywords:** skin cancer, cutaneous squamous cell carcinoma (cSCC), actinic keratosis (ak), genetic landscape, tumor microenvironment, mutations

## Abstract

**Simple Summary:**

Cutaneous squamous cell carcinoma (cSCC) is a complex disease arising from an interplay of UV-induced DNA damage, genetic mutations, and alterations in the tumor microenvironment. While most cSCC cases can be treated with surgery, metastatic and locally advanced forms have a significantly worse prognosis and limited treatment options. The high mutational burden in cSCC makes it challenging to identify true driver alterations. Beyond genetics, the tumor microenvironment and its interaction with the immune system play a critical role in cSCC progression, evidenced by differences observed between immunocompromised and immunocompetent patients. In recent years, new therapeutic approaches have emerged, including immunotherapy and EGFR inhibitors, which have shown efficacy in treating advanced cSCC. Understanding the underlying mechanisms driving cSCC is crucial for developing novel and more effective therapies. This review aims to provide a comprehensive overview of the mutational landscape and microenvironmental factors in cSCC.

**Abstract:**

Cutaneous squamous cell carcinoma (cSCC) manifests through the complex interactions of UV-induced DNA damage, genetic mutations, and alterations in the tumor microenvironment. A high mutational burden is present in cSCC, as well as both cSCC precursors and normal skin, making driver genes difficult to differentiate. Despite this, several key driver genes have been identified, including TP53, the NOTCH family, CDKN2A, PIK3CA, and EGFR. In addition to mutations, the tumor microenvironment and the manipulation and evasion of the immune system play a critical role in cSCC progression. Novel therapeutic approaches, such as immunotherapy and EGFR inhibitors, have been used to target these dysregulations, and have shown promise in treating advanced cSCC cases, emphasizing the need for targeted interventions considering both genetic and microenvironmental factors for improved patient outcomes.

## 1. Introduction

Cutaneous squamous cell carcinoma (cSCC) is the second most common subtype of non-melanoma skin cancer, as it is responsible for 20% of all skin cancers [1,2]. Major risk factors include ultraviolet (UV) radiation exposure, male sex, pale complexion, xeroderma pigmentosum, and immunosuppression [2,3,4,5,6,7]. Treatment depends on the level of aggressiveness and metastatic status, with non-metastatic cases usually successfully removed via surgical excision [6]. Metastatic cSCC, which accounts for only 5% of cases, is associated with a worse prognosis and often requires a combinatory treatment of surgery, radiation, and systemic therapy [2,6,8]. 

The current understanding of cSCC development is multifaceted, with the tumorigenesis associated with it contributing to a disruption of epidermal homeostasis, stemming from alterations at various points of the development process; these alterations include UV-induced DNA damage, gene mutations, and tumor microenvironment changes [9,10]. The recent success in the treatment of cSCC with immune checkpoint inhibitors also demonstrates the important interplay between cSCC and the tumor immune microenvironment [11,12]. 

This review presents an overview of the literature on cSCC from a genetic and tissue microenvironmental perspective to improve our current understanding of this disease and highlight potential novel therapeutic targets for future study. 

## 2. Genetic Landscape

### 2.1. UV-Signature Mutations

Ultraviolet exposure, specifically UVB exposure, is the most important, preventable risk factor in the development of cSCC, as it induces DNA mutations within the skin [8,13]. Normally, these mutations are repaired via a process known as nucleotide excision repair (NER), but when NER process is defective, cSCC development can occur [13]. The most frequent UV-signature mutations are C > T substitution mutations at dipyrimidine sites, often accompanied by CC > TT dinucleotide substitutions on the non-transcribed strand of many genes [1,14,15]. C > A (G > T) is another described mutational signature seen in cSCC with low mutational burden [1,15]. Recent research has suggested a novel UV mutational signature, revealing that T > C substitutions are also a frequent result of UVB exposure, with 5’-ATT-3’ and 5′-ATG-3′ as reported sequence motifs [16].

### 2.2. Mutations in Normal Skin 

Identifying UV signatures alone is insufficient in understanding the trajectory from cSCC to tumorigenesis. Interestingly, many of mutations in cSCC are also seen in normal sun-exposed skin [15]. Genes from the NOTCH family (NOTCH1, NOTCH2, and NOTCH3) are frequently mutated in normal skin, with alterations in NOTCH genes having an average of 83 driver mutations per square centimeter of normal skin, resulting in significant protein alterations [15]. TP53 is also frequently mutated in normal skin, with a rate of 9.5 driver mutations per square centimeter of skin [15]. Furthermore, mutations in FAT1 and well-known oncogenes like KRAS, NRAS, and HRAS have also been identified in normal skin [15]. 

This high mutation occurrence in normal skin makes differentiating somatic driver mutations from passenger mutations quite difficult [1,5]. Other limitations include tumor heterogeneity, neoplastic cell content, mean sequencing coverage, and variable sequencing coverage across tumors [5]. Fortunately, these limitations can be addressed by using large cohort sizes [1], conducting meta-analysis [5], having strict quality control measures, and understanding the mutational burdens in normal skin [5,15].

### 2.3. Hereditary Mutations

While most cSCCs arise from somatic mutations, certain hereditary diseases can also contribute to the development of cSCC. Xeroderma pigmentosum (XP) is a genetic condition known to predispose patients to cSCC, basal cell carcinoma (BCC), and melanoma [3,17,18]. XP is an autosomal-recessive disorder that causes extreme sensitivity to UV exposure, due to mutations in the NER pathway, which hinder the DNA’s ability to repair itself after UV damage [5,18]. As a result, XP patients have an extremely high risk for developing skin cancer, with patients under 20 having a 10,000-fold increased risk of developing cSCCs and BCCs and a 2,000-fold increase for melanoma [3]. 

### 2.4. Mutations in Pre-Cancer

The primary cSCC precursor is actinic keratosis (AK), an in situ neoplasm that originates from mutated clonal DNA in keratinocytes [19,20,21]. AKs manifest as macules, papules, or hyperkeratotic plaques that are identified via visual and palpation inspections [20]. Both AK and cSCC share the same risk factors, the most important being UV exposure, as well as male sex, fair skin, and old age [19,20]. However, only some AK cases progress into cSCC, and the exact pathway to malignancy is not well understood [20,21]. 

The ability to define clear genetic criteria for the malignant transformation of AK to cSCC is limited by the high-UV mutational burden [20,21]. Both cSCC and AK demonstrate many genetic similarities, including mutations within driver genes and mutational signatures [21]. TP53 is renowned for being an early mutational event in both AK and cSCC, usually as nonsense or missense mutations with specific hotspots R248W and G245D found in normal sun-exposed skin [1,5,8,19,20,21,22,23,24]. Other genetic alterations in genes NOTCH1, NOTCH2, FAT1, and KMT2C and mutational signatures C > T and CC > TT have been found in both AK and cSCC; however, whether or not these mutated genes and UV signatures can reliably predict the trajectory from AK to cSCC progression is still being investigated [21]. One study found that cSCC is most associated with common and hypertrophic AK types, with low p53 immunopositivity predicting if common-type AK will develop into cSCC [19]. While 65% of SCCs are derived from AKs, only about 0.6–2.57% of AK cases progress into cSCC, partly because AK can regress [25,26]. 

Although, AK and cSCC are very similar in their mutational burden values, 43.5 mutations per megabase DNA and 50 mutations per megabase DNA respectively, cSCC experiences more intra-sample heterogeneity than AK [21,27]. They also differ in terms of the many pathways they involve, which are activated in cSCC but not activated in AK, such as the PI3K/AKT/mTOR and RAS/RAF/MEK/ERK pathways [28,29,30,31,32]. Additionally, immune system signaling pathways like those for TGFβ, adipocytokine, GnRH, and insulin signaling are more highly mutated in cSCC than in AK [21]. 

### 2.5. Major Driver Pathways of cSCC

**Figure 1:**
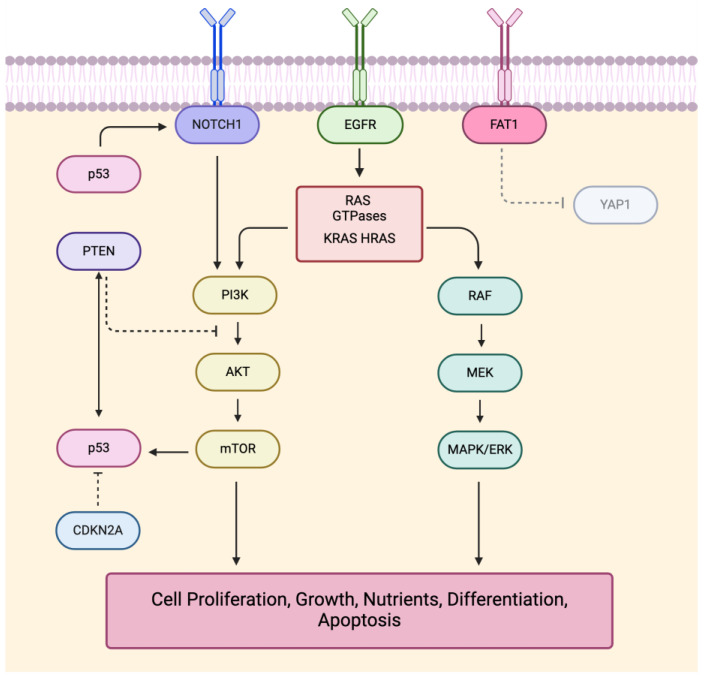
Visualization of the pathway and driver gene interactions in cSCC. Figure references [1,5,27,28,29,31,34,35,36,37,38,39,40,41,42,43,44,45,46,47].

Despite the high mutational load of cSCC and the challenges in determining driver genes within cSCC, many of these genes have been identified, as seen in Table 1. The well-known cSCC drivers are also famously mutated in many other cancers; these include TP53, NOTCH, CDKN2A, PIK3CA, FAT1, RAS, EGFR, and HRAS [7,8,21,23,31,33,34]. These genes often interact with each other in pathways strongly activated in cSCC, such as the PI3/AKT/mTOR, MAPK/AKT, and RAS/RAF/MEK/ERK pathways, and their interactions can be seen in Figure 1 [7,28,29,31]. While many of these driver genes play a role in tumor suppression, and the interplay of their mutations additively contributes to tumorigenesis, the exact mechanisms through which they contribute to malignancy are not fully understood. 

#### 2.5.1. TP53

TP53 is one of the most frequently mutated driver genes in cSCC, as well as in almost all human cancers [10,21,27,51]. Its protein product p53 is a tumor suppressor protein that regulates cell cycle arrest, genome stability, DNA damage repair, senescence, and apoptosis [52,53]. Within cSCC, TP53 mutations are very heterogenous, including point mutations, missense mutations, and nonsense mutations that can result in either gains in or a loss of function of the p53 protein [54]. Some TP53 hotspots have been reported in cSCC, at R248W and R248Q, which are conserved arginine locations at the DNA binding interface [54,55,56]. TP53 mutations are suggested to be the initiating mutations in UV-induced cSCC, occurring in 54–95% of cases [7,57]. TP53 interacts with other well-known cSCC driver genes such as CDKN2A, NOTCH1, and NOTCH2, and its mutation has been linked to UV exposure and C > T/CC > TT UV mutational signatures [10,57,58]. Despite its definite role in cSCC tumorigenesis, TP53 mutation alone has not been proven to predict malignancy [19,59]. While many cases of AK have mutations in the TP53 gene, most never progress into cSCCs, making it challenging to consider TP53 a predictive biomarker [19,25]. In addition, TP53 is also mutated in normal skin, meaning that TP53 alone cannot lead to cSCC development, and its interplay with other driver genes must be explored further [15,57].

#### 2.5.2. NOTCH Family

The NOTCH family comprises tumor suppressor genes, particularly NOTCH1, NOTCH2, and NOTCH3, which are mutated in cSCC and encode receptors essential for cell differentiation and cell cycle arrest [60,61,62]. NOTCH1 is the most common driver gene mutated in normal skin, affecting 20% of skin cells [15]. In cSCC, NOTCH1 and NOTCH2 mutations are loss-of-function mutations that occur in around 50–80% of cases [1,5,27,62]. Although NOTCH1 and NOTCH2 are paralogs and present within the same signaling pathway, they are present in different layers of the epidermis, with their differing signals resulting in different cancer prognoses [60,63,64,65,66]. NOTCH1 expression is mediated by p53, and like TP53 mutations, NOTCH1 mutations are believed to occur early on during cSCC [15,60,61,62].

#### 2.5.3. CDKN2A

CDKN2A, or cyclin-dependent kinase inhibitor 2A, is a protein-coding gene that has functionality in G1 cell cycle control [67,68,69]. CDKN2A encodes two proteins, p16^INK4a^ and p14^ARF^, which are negative regulators of cell proliferation and interact with p53 as part of the cell regulation pathway [60,70]. Like TP53, CDKN2A mutations are present early on in cSCC tumorigenesis [68,70]. In cases of other squamous cell carcinomas, such as head and neck squamous cell carcinoma (HNSCC), patients with severe mutations in both TP53 and CDKN2A have a decreased survival rate and more aggressive cancer forms, suggesting that CDKN2A plays a role in suppressing metastasis [68,70]. This finding was also mirrored in cSCCs, suggesting that CDKN2A mutational presence in a metastatic tumor can predict the prognosis of metastatic cSCC and SCCs [68,70].

#### 2.5.4. PIK3CA Pathway—AKT, mTOR, MAPK, PTEN

The PIK3CA gene encodes phosphoinositide 3-kinases, catalytic enzymes that play a role in the PI3K/AKT/mTOR pathway [29]. The PI3K/AKT/mTOR pathway is consistently activated in cSCC but not in its precursor, AK, suggesting that its dysregulation may be responsible for pre-cancer to cSCC transition [7,28,29,31]. Dysregulation of the pathway can occur at various points: when activated by PI3KCA mutations, upon UV exposure, or after a PTEN loss of function mutation [7,28,29]. PTEN, a tumor suppressor phosphatase, negatively regulates the PI3K/AKT/mTOR pathway by inhibiting PI3K from converting PIP2 into PIP3 [7,29,71]. PTEN has been reported to be mutated in cases of cSCC [5,27,31].

Once PIP2 converts into PIP3, it activates AKT, a serine/threonine protein kinase that regulates terminal differentiation, cell proliferation, the cell cycle, and cell migration through its signaling [7,29,71]. AKT has multiple isoforms that perform independent functions: Akt1, Akt2, and Akt3 [29]. Akt1 is expressed in the top layers of the epidermis and reinforces the epidermal barrier [29,72]. Akt2 is found in the lower layers, is responsible for wound healing and proliferation, and is suggested to contribute more to cSCC tumor progression [29,72,73]. Studies on the two isotopes have inferred that during cSCC progression, Akt1 expression is downregulated while Akt2 expression is upregulated [72]. AKT also plays a critical role in regulation through its intermediate position in the PI3K/AKT/mTOR pathway [7,29,71].

mTOR activation allows for elevated levels of proteins, nucleotides, and lipids, making the protein kinase imperative in sustaining tumor growth [74]. Its position in the PI3K/AKT/mTOR pathway has made it a potential therapeutic target, and mTOR inhibitors, such as rapamycin, have been used to treat a variety of conditions and various other non-cSCC cancers [75].

#### 2.5.5. MAPK(ERK)—RAS/RAF/MEK/ERK Pathway

MAPKs, or mitogen-activated protein kinases, also known as extracellular signal regulated kinases (ERKs), are responsible for signaling roles in many cellular functions, such as cell proliferation, survival, apoptosis, migration, differentiation, and cell cycle regulation [28,29,30,32]. MAPK or ERK is the final factor in the RAS/RAF/MEK/ERK pathway, and is commonly dysregulated across varying cancers [76]. Many studies have found MAPK pathways being activated after UV irradiation and significantly activated in cSCC as opposed to AK, suggesting that the MAPK pathway is critical in the transition from AK to cSCC, possibly through its role in the apoptotic response [28,29,30,32]. Thus, MAPK has been made an ideal target for therapeutic approaches, with studies showing its inhibition in response to photodynamic therapy [32].

#### 2.5.6. RAS Genes: HRAS, NRAS, and KRAS

RAS genes comprise a family of GTPase proteins of three gene isoforms: HRAS, NRAS, and KRAS [76]. Among the three, HRAS is most frequently mutated and associated with cSCC, followed by KRAS, and then in a few cases NRAS [1,5,7,36]. RAS GTPases are upstream of many important pathways in cellular functions, including the PIK3CA and the RAF/MEK/ERK pathways [28]. Upstream of the RAS genes are various receptor tyrosine kinases, one of which is EGFR [77]. When both RAS and EGFR are mutated, there is considerable dysregulation, and the use of EGFR inhibitors becomes ineffective [77]. RAS itself has been proposed as a potential therapeutic target in cSCC [78]. Using salirasib, an anti-RAS drug, L.Li et al., aimed to suppress RAS/RAF/MEK/ERK signaling and in turn decrease the expression of cellular proliferation and regulatory processes; however, the anti-RAS drug was only partially successful in inhibiting the pathway in cell culture [78].

#### 2.5.7. EGFR

EGFR, an epidermal growth factor receptor, is involved in cellular processes such as tissue regeneration, construction, homeostasis, cellular apoptosis, proliferation, and differentiation [28,35,79]. In skin keratinocytes, EGFR is highly mitogenic and activated by UV exposure [79]. In an experiment with EGFR knockout and wild-type mice, El-Abaseri et al., found that after UV exposure, wild-type mice showed cell proliferation that had increased to levels 3× higher than in those in EGFR knockout mice, and that UV-exposed EGFR knockout mice expressed higher levels of apoptosis [79]. 

Mutations in EGFR resulting in upregulation can be rather disruptive as they have the potential to trickle down to the downstream pathways, further encouraging tumor progression [35]. Within cSCC, EGFR has been reported to have both activating mutations and aberrant overexpression [31,35]. Some studies have found the high epidermal expression of EGFR to be associated with poor prognosis due to increased metastasis and migration to the lymph node [8,35,80,81]. However, other research disputes EGFRs’ correlation with poor outcomes in cSCC, leaving its exact effect on prognosis not understood [80,82,83]. EGFR is upstream of many pathways such as the RAS/RAF/MEK/ERK, PI3K/AKT/mTOR, and JAK/STAT pathways, making its activation very influential and an ideal target for therapeutic interventions [7,28,31,35]. Currently, EGFR inhibitors are available as therapeutic options for select cSCC patients [82]. 

#### 2.5.8. FAT1

Traditionally downregulated due to a loss of function mutation, FAT1 is a protocadherin known to contribute to cellular migration, mitochondrial respiration, and cell proliferation in normal tissues [27,84,85,86]. In cancer, the FAT1 mutation promotes metastasis, invasiveness, malignant progression, and tumor initiation [1,27,84,85,86]. FAT1 mutations are also found in normal skin but at a much lower rate than other driver genes are found, in only around 3–5% of the skin cells studied [33]. In cSCC models, it was found that FAT1 deletions promote tumor initiation and progression, and increase YAP1 expression, promoting a mesenchymal state within the tumor tissue [86]. In other SCCs, such as esophageal squamous cell carcinoma (ESCC) and hepatocellular carcinoma (HCC), FAT1 was found to be a negative regulator of the epithelial–mesenchymal transition (EMT), with the loss of function of FAT1 promoting EMT, cell growth, migration, and invasion [86,87,88]. This suggests a mechanism in which FAT1 mutations promote tumorigenesis in cSCC and other cancers, but still, more research is needed to investigate this further [86,89].

### 2.6. Non-Coding Portion of Genome

Mutations in cSCC have also been detected in the non-coding portion of the genome, including telomeric regions of genes [90,91]. Encoded by the hTERT gene, telomerase prevents telomere shortening, but when mutated, telomerase can permit cells to replicate indefinitely and contribute to carcinogenesis [91]. The mutations are present in the TERT promoter region rather than in the hTERT gene sequence [7,44,90]. In cSCC, TERT promoter mutations occur in up to 30–70% of cSCC tumors [7,8,44,91,92,93]. Interestingly, telomere shortening has been reported at almost all stages of cSCC progression, with the telomere length in cSCC found to be significantly shorter than that in its precursor AK [91,92]. According to Campos et al., invasive cSCCs have more mutations in the TERT promoter than non-invasive cSCCs do, and these mutations can independently predict the local recurrence of cSCC [44].

Other alterations within non-coding portions of the genome that have been reported in cSCC include long non-coding RNAs (lncRNAs), circular RNAs (circRNAs), and microRNAs (miRNAs) [94,95]. Long non-coding RNAs (lncRNAs), regulatory RNAs with over 200 nucleotides, and circular RNAs (circRNAs), RNA back-spliced into a continuous loop, have been identified in cSCC as novel regulators of gene expression [94]. Mahapatra et al., detected 908 lncRNAs and 55 circRNAs to be differentially expressed in cSCC [94]. Among these are known oncogenic lncRNAs: PVT1, LUCAT1, and CASC9 [94]. Novel skin-specific lncRNAs were also found to be dysregulated [94]. A global reduction in circRNA was seen in cSCC as opposed to normal skin [94].

Many miRNAs have been reported to be dysregulated in cSCC, including the upregulation of Onco-miRNAs miRNA-21, miRNA-31, miRNA-135b, and miRNA-365 [96]. Interestingly, many miRNA target genes are within the known cSCC pathways, mediating genes like PTEN, KRAS, and ERK [97,98,99,100]. Specifically, PTEN is a frequent target of many miRNAs types such as 142, 221, and 21 [95,97,101,102]. 

### 2.7. Novel cSCC Driver Genes

In the last ten years, increased efforts to genotype and analyze the mutational landscape of metastatic, aggressive, advanced, and traditional cSCC have allowed for various potential driver genes to be discovered. Table 2 lists genes that have been recognized among several primary research papers as relevantly mutated and/or nominated as novel driver genes across the cSCC subtypes. The following genes were nominated in three primary research papers: CCND1, CREBBP, EP300, ERBB4, EZH2, FGFR3, KMT2C, KMT2D, and TGFBR2 [1,5,31,37,38,39,40,41,42,50]. Additionally, genes that were nominated in two primary sources include AJUBA, ARID2, ATM, BRAF, BRCA2, CASP8, KNSTRN, LRP1B, NFE2L2, CARD11, MYC, and RIPK4 [1,5,31,37,39,40,42,43,103,104]. Of the genes nominated, many are known contributors in other cancers [1,31]. The genes RIPK4, CCND1, CASP8, and MYC are also reported to be commonly mutated or dysregulated in HNSCC [31]. BRAF mutations, found at very low occurrence rates in cSCC, are linked with several different cancers, such as metastatic colorectal cancer, thyroid cancer, and melanoma [31,105,106]. 

While there are many limitations in uncovering these novel drivers, the genetic analysis and mutation rates of these drivers are crucial in understanding cSCC development, and in turn can aid in finding new therapeutic drug targets. 

## 3. Tumor Microenvironment

As previously mentioned, there is still a lack of clear understanding in the process of the malignant transformation of cSCC, as mutational landscapes can be similar between normal skin, pre-cancers, and invasive cSCC. Therefore, understanding other external factors such as the tumor microenvironment is critical to elucidating mechanisms of cSCC pathogenesis. As solid organ transplant recipients (OTRs) are at a 50–100-fold increased risk for the development of cSCC, their immunosuppressed microenvironment is a clear contributor to cSCC pathogenesis [122,123,124,125]. Generally, OTRs have decreased amounts of immune infiltrates, including lower B-cell infiltrates, as well as a variety of T-cell types (CD4+, CD4+T helper 1, CD8+, cytotoxic CD8+, Naive CD8+, etc.) [122,123,124,125]. While the risk for skin cancers is most significant, it is understood that OTR patients are also at an increased risk of developing other cancers, such as Non-Hodgkin’s lymphoma, kidney cancer, liver cancer, and Kaposi’s sarcoma [126,127]. Therefore, herein, we also explore the current understanding of tumorigenesis beyond the genome, focusing on the extracellular matrix, stromal cells, and immune cells [128,129].

### 3.1. The Extracellular Matrix and MMPs

The extracellular matrix (ECM) is a critical cell microenvironment component. While non-cellular itself, it provides cells with structure and an opportunity to interact with each other through extracellular signaling [130]. During cSCC progression, ECM remodeling occurs, often forming a dense fibrosis around solid tumors [131,132,133]. These modifications can be attributed to proteolytic enzymes, such as matrix metalloproteinases (MMPs) and A disintegrin and metalloproteinase with thrombospondin motifs (ADAMTS) [130,134,135]. With their ability to break down proteins, MMPs and ADAMTS can completely denature and reorganize the ECM to facilitate the development of cSCC [134,135]. 

#### 3.1.1. MMPs

MMPs are found to be upregulated in cSCC, and their increased expression is deemed a significant biomarker in predicting prognosis, aggressiveness, and metastasis [130,133,134]. MMP-13 expression has been proposed to detect the level of invasiveness of cSCC and can be used to monitor progression [133]. MMP-9 and MMP-11 expression encourages matrix turnover, while MMP-10 enhances this process and angiogenesis, facilitating metastasis in cSCC [134,136,137,138]. Additionally, MMP-7 expression was found in the invasive front of cSCC tumors, suggesting a role in tumor progression [139]. 

#### 3.1.2. Collagen

Collagen is a fibrous protein that makes up the majority of the ECM [130,134]. The 28 types of collagens identified to date are versatile and abundant within the skin, making them candidates for cancer complicity [140]. In cSCC, collagen XVII is expressed widely by tumor cells at high expression levels not seen in normal skin or cSCC precursors [141]. Another collagen, collagen VII, suppresses TGFβ signaling and angiogenesis in cSCC [142]. In vivo, the knockout of collagen VII promoted angiogenesis, with collagen VII treatment decreasing tumor angiogenesis [142]. Less is known about collagen XV’s contribution to cSCC tumorigenesis, but it is highly expressed in cSCC tumors and particularly abundant in tumor stromal cells, suggesting that collagen XV may be linked to cancer-associated fibroblasts [142]. 

#### 3.1.3. Stromal Cells 

Stromal cells normally aid in the regeneration of tissues, immune responses, ECM remodeling, and tissue homeostasis [143,144]. In the tumor microenvironment, stromal cells known as cancer-associated fibroblasts (CAFs) promote tumorigenesis by providing growth factors and nutrients to the newly formed tumor and blood vessels [145]. In cSCC specifically, Martínez-Nieto et al., found that integrin α11β1 was upregulated in cancer-associated fibroblasts and other human neoplasms [129]. Additionally, α11-deficient cells hindered stromal cells’ ability to differentiate into tumor-promoting CAFs, and as a result, the mice experimented on had decreased tumor cell proliferation, development, and burden [129]. This suggests that α11 expression in stromal cells helps cSCC tumors to progress to malignant states [129]. Studies have also found that the secretion of growth factor TGFβ1 by CAFs contributes to increased resistance to photodynamic therapy, signifying that CAF-secreted TGFβ1 can be used as a biomarker to determine the effectiveness of photodynamic therapy in cSCC patients [144].

### 3.2. Immune Cell Populations

#### 3.2.1. Macrophages

In normal tissues, macrophages are an integral part of the immune system, removing threats to the immune system through phagocytosis [146]. In cancer tissue, tumor-associated macrophages (TAMs) can alter both the tumor and immune microenvironment [138].

TAMs are more abundant in cSCC than normal skin, and studies show that these TAMs promote tumor growth and metastasis [138,147]. TAMs can produce VEGF-C, vascular endothelial growth factor to stimulate growth, lymph angiogenesis, and metastasis [138,148,149]. They also contribute to cSCC tumorigenesis through their generation and secretion of MMPs, specifically MMP9 and MMP11 [138].

Within the cSCC microenvironment, TAMs are in heterogeneous activation states, either M1 or M2, with some being bi-activated, expressing both M1 and M2 markers [138]. TAMs under M2 activation tend to promote tumorigenesis, while M1 macrophages promote the pro-inflammatory response and induce the anti-tumor immune response [148,150]. Notably, OTR patients demonstrate similar levels of TAM infiltration but significantly fewer M2-activated TAMs [151]. While more recent research suggests that TAMs are not strictly adherent to M1/M2 states and can be alternatively defined based on their effect on the TME as either pro- or anti-inflammatory [152], most studies on cSCC have focused on traditional M1/M2 classifications.

In general, an increase in TAMs, especially M2 ones, is associated with poor prognosis across many other cancers, such as breast cancer, oral cutaneous cell carcinoma, and prostate [138,153,154,155,156,157,158]. TAMs are a potential target in immunotherapy, as the repolarization of TAMs into anti-tumor macrophages could be used to slow tumor progression [159].

#### 3.2.2. T-Cells

Limited studies have examined T-cell populations within cSCC, focusing mainly on the immune microenvironment differences between cSCC and OTR cSCC. The latter is generally more aggressive, implying that microenvironmental differences across immune states could explain the variation in responses to immunotherapy. 

Cytotoxic T-cells from OTR patients respond differently than those in regular cSCC patients, favoring the production of cytokine IL-22 [160]. Both cSCC and OTR cSCC show significantly higher numbers of CD3+, T-Lymphocytes, and CD8+, cytotoxic T-cells, as opposed to normal skin, with cSCC having significantly more of these cells than OTR cSCC [122,160]. Topographically, in both cSCC and OTR cSCC, CD3+ and CD8+ T-cells are located primarily in peritumoral regions as opposed to within the tumor nests [160].

In cancer, T-regulatory (Treg) cells can suppress anti-tumor responses, with higher proportions of tumor-infiltrating Treg cells correlating with poorer prognosis [161,162,163,164,165]. This pattern has also been noted in cSCC [165,166]. Tregs are more abundant in cSCC sites than in the peripheral blood, contributing to metastasis through their suppression of effector T-cells and IFN-γ expression [165]. While Treg cells are reported at similar levels in cSCC and OTR cSCC, both are at significantly higher levels than in normal skin [122,160]. The Treg-to-cytotoxic T-cell ratio is higher in OTR cSCC than in cSCC, potentially promoting immune evasion [122,160].

cSCC patients had higher levels of naïve T-cells than OTR cSCC patients did, but shared similar numbers of exhausted T-cells [122]. Within the same patient, high intertumoral heterogeneity of T-cell populations can be present [122]. One report showed that, in a single OTR patient, a forehead tumor demonstrated mainly cytotoxic T-cells, while a tumor from the neck predominantly showed exhausted T-cells [122]. In general, exhausted and effector T-cells had the highest proliferation capacity across the T-cell subsets, with exhausted CD4+ and CD8+ cells expressing inhibitory receptors and exhaustion markers [167].

#### 3.2.3. Natural Killer Cells

Natural killer (NK) cells, as part of the innate immune system, can kill tumor cells without antigen priming or major histocompatibility complex (MHC) interactions, and without harming normal cells [168]. Generally, in cancer, NK cell functions are diminished [168,169]. Higher rates of cSCC are seen in patients with functional NK cell deficiencies, implying that NK cells play a role in inhibiting cSCC progression [170,171]. 

Studies using Nfil3^−/−^ knockout mice demonstrated NK cells’ critical role in early cSCC tumorigenesis, as these mice developed significantly more papillomas and pre-cancerous protrusions, and had increased tumor growth [169]. When these tumor-bearing mice were treated with NK cells, the tumor size decreased to levels that were near those of wild-type Nfil3^+/+^ [169]. However, NK cells isolated from these tumors showed reduced cytotoxicity, IFN-γ production, and secretion [169]. 

NK cells’ pivotal role and dysfunction in the TME has prompted research into their therapeutic potential. A recent preclinical model explored if the transplantation of NK cells from healthy donors into non-autologous patients could serve as a potential therapeutic approach [168]. In vitro, the co-incubation of SCC-13 cells with healthy NK cells resulted in a dose-dependent reduction in the size of spheroids and in the number of tumor cells [168]. In vivo, mice with SCC-13 xenograft tumors treated with NK cells saw a 70% reduction in tumor volume at three weeks [168]. In both in vivo and in vitro models, a reduction in YAP1 and MEK1/2-P levels occurred, demonstrating that the healthy NK cells were able to induce apoptosis [168].

#### 3.2.4. Neutrophils 

In vivo and in vitro experiments have found that tumor-associated neutrophils (TANs) are present in both pre-cancerous and cancerous cSCC lesions, with 30–80% of neutrophils present exhibiting the TAN phenotype [172]. In general, neutrophils were localized to the pre-cancerous and cancerous regions as opposed to the surrounding skin, with significantly higher percentages in tumors as opposed to the papillomas [172]. The orthotopic implantation of cell line mSCC38 in mice also supported these findings, with neutrophil frequency being positively correlated to the mSCC38 tumor volume and having decreased frequency in the surrounding skin [172]. 

The functions of TANs evolve with the carcinogenesis stages. In pre-cancerous stages they contribute to ECM remodeling and angiogenesis [172]. In cancerous stages, TANs actively suppress immune responses by inhibiting CD8+ T-cell responses and upregulating PD-L1 [172]. In vivo depletion of neutrophils significantly increased the proliferation of CD8+ T-cells and elevated IFN-γ levels, further supporting the immunosuppressive role of TANs in cSCC [172].

Clinically, elevated neutrophil counts have been associated with increased tumor thickness and poor overall survival in cSCC patients, warranting further investigation of neutrophils as a potential prognostic marker [173]. 

## 4. Novel Therapeutics 

With the current understanding of driver genes of cSCC and the tumor microenvironment in cSCC, certain therapeutic approaches have been pursued. In recent years, novel therapies, including immune checkpoint inhibitors and EGFR inhibitors, have demonstrated promising results for the treatment of cSCC, particularly in unresectable or recurrent/metastatic disease (Table 3). While overall response rates (ORRs) to these treatments can range between 8% and 63%, patients who do not respond and OTR patients who cannot receive immune checkpoint inhibitors are in need of novel therapies. 

### 4.1. Immunotherapy

Immunotherapy has revolutionized cancer treatment in the past decade. These therapies work by upregulating immune activation to produce an anti-tumor response, specifically through monoclonal antibodies inhibiting immune checkpoints CTLA-4 or PD-1/PD-L1 [182]. In the last 6 years, two anti-PD1 immunotherapy drugs have been approved by the FDA for cSCC treatment: cemiplimab and pembrolizumab [11,183,184]. 

In 2018, cemiplimab became the first FDA-approved PD-1 checkpoint immune inhibitor for metastatic or local advanced cSCC [185]. This approval stemmed from phase I and phase II clinical trials conducted by Migden et al., which demonstrated durable and promising responses in about half of the patients [11]. In a phase II trial, 79 advanced cSCC patients not amenable to surgery or radiation were treated with cemiplimab [12]. This showed a high disease control rate of 79% in patients with at least stable disease, with 31% showing partial response and 13% showing complete response [12]. Following the FDA approval of cemiplimab in 2018, trials with pembrolizumab demonstrated similar efficacy in cSCC, leading to subsequent approval in 2020 [83,174]. Subsequently, a recent phase II neoadjuvant study for previously untreated resectable cSCC demonstrated that administering 2-4 cycles of cemiplimab before surgery led to the achievement of a complete pathologic response in 51% of patients and a major pathologic response in 63% (<10% viable tumor) [12]. Given these high pathologic response rates, future studies are investigating the long-term impact of neoadjuvant treatment as well as the role of adjuvant immunotherapy after surgery. In melanoma, the addition of neoadjuvant immunotherapy before surgery significantly improved outcomes compared to using immunotherapy in the adjuvant setting after surgery, demonstrating the potential advantage of the early introduction of these treatments before the removal of the primary tumor [186].

### 4.2. EGFR Inhibitors

When patients are elderly, or immunocompromised, and surgery and radiation therapy fail, the use of EGFR inhibitors is another treatment option for cSCC. As mentioned previously, EGFR dysregulation is a known characteristic within cSCC; however, the exact source of EGFR dysregulation is an important determinant in the success of EGFR inhibitor therapy. While EGFR dysregulation is found in cSCC patients, downstream RAS mutations can limit drug efficacy [31,82,83]. Multiple agents targeting EGFR are available, including monoclonal antibodies such as cetuximab and panitumumab, and small-molecule inhibitors such as gefitinib and erlotinib. The effectiveness of these inhibitors in cSCC are being investigated, both as a monotherapy and in addition to radiation or immunotherapy [83,187,188]. 

#### 4.2.1. EGFR Monotherapy

Usually, when immunotherapy is not an option for patients, EGFR monotherapy can be considered. In a 2011 phase II clinical study by Maubec et al., 36 patients with unresectable cSCC received cetuximab as a monotherapy [175]. At 6 weeks, 68% of the patients were able to achieve stable disease [175]. However, only 6% achieved a CR, 26% had a PR, and 19% had progressed disease [175]. In a retrospective analysis conducted by Marin-Acevedo et al., cetuximab showed a 64% ORR (*n* = 11) in patients after failing anti-PD1 therapy, and an 80% ORR (*n* = 10) in anti-PD1-naïve patients [187]. While promising, it is important to note that retrospective studies are limited by their selection bias, small sample size, and heterogenous population.

Panitumumab has also shown promise as a monotherapy for cSCC. In the phase II (*n* = 16) study conducted by Foote et al., it was found that patients with locally advanced, metastatic, or recurrent cSCC, previously treated with either radiotherapy (*n* = 14) and/or cytotoxic chemotherapy (*n* = 7), saw an overall response rate of 31%, with 6 of the 16 successfully achieving stable disease [176]. Phase II clinical trials have also been conducted to investigate the effect of erlotinib and gefitinib on cSCC; however, they were only reported to have modest responses [177,178]. It is important to note that these studies are limited by their sample size; thus, more research is needed to further confirm the positive results.

#### 4.2.2. EGFR Inhibitors with Radiation 

Currently, there have only been retrospective studies on the efficacy of EGFR and radiation combination therapy for cSCC. Most recently, a retrospective case series of 18 patients with locally advanced cSCC found a 55.5% CR in combinatory radiation and cetuximab therapy [189]. Nevertheless, high ORR in other SCCs, such as HNSCC, suggest that this combinatory therapy could also be effective in cSCC. In locally advanced HNSCC, Bonner et al., saw improved overall survival when cetuximab was combined with radiation (a median survival of 49 months) compared to that when using radiotherapy alone (29.3 months) [190].

#### 4.2.3. EGFR Inhibitors with Immunotherapy 

Combinatory therapy with cetuximab and anti-PD1 inhibitors still needs to be explored further, with retrospective and case reports suggesting promise in its efficacy. In Hsu et al., panitumumab was used in combination with anti-PD1 in three patients who had progressed on anti-PD1 inhibitors, with all patients achieving durable CR [191]. 

While limited data exist on the combination of EGFR inhibitors with immunotherapy in cSCC, the use of this combinatory treatment in other squamous cell carcinomas suggests that it could also be successful in cSCC [180,181]. Sacco et al., conducted a phase II trial treating recurrent or metastatic HNSCC with cetuximab followed by pembrolizumab [180]. After 6 months, there was an ORR of 45% [180]. In another phase II trial treating recurrent/metastatic HNSCC patients with cetuximab followed by nivolumab, Chung et al., found an ORR of 23% in patients with prior treatment for recurrent/metastatic disease and 33% in patients for which therapy was first line for recurrent/metastatic disease [181]. 

## 5. Conclusions

The high mutational burden in cSCC complicates our understanding of its biological underpinnings. This complexity makes it challenging to differentiate between passenger alterations present in normal skin and premalignant lesions, and to identify the driver alterations that contribute to carcinogenesis. The tumor microenvironment also contributes significantly to tumor progression, and the interplay between the immune system has allowed for the success of immunotherapies in the treatment of cSCC. For patients who do not respond to immunotherapy, novel targeted treatments are under investigation in targeting pathways including EGFR inhibition. Additional therapeutic opportunities may be available in targeting other pathways such as mTOR inhibitors, photodynamic therapy, or cancer-associated fibroblasts and tumor-associated macrophages. Continued research integrating genomic profiling, microenvironment analysis, and novel therapeutic approaches holds promise for improving outcomes in cSCC patients.

## Figures and Tables

**Table 1:** Major validated driver genes and pathways in cSCC; Genes and pathways cited in four or more primary papers as mutated or dysregulated in cSCC, and consistently validated as key components in cSCC tumorigenesis. Description references [1,5,27,28,29,31,34,35,36,37,38,39,40,41,42,43,44,45,46,47].

Gene(s)	General Description	References
TP53	Tumor protein p53; regulates genome stability, cell proliferation, and apoptosis	[1,5,27,31,36,37,40,45]
NOTCH 1,2,3	Notch receptor; regulates cell signaling, differentiation, cell cycle arrest, and apoptosis	[1,5,27,31,36,37,45]
CDKN2A	Cyclin-dependent kinase inhibitor 2A; regulates genomic stability, Cell cycle control, and cell proliferation	[1,5,27,31,40,48]
RAS/RAF/MEK/MAPK(ERK) Pathway	Regulates cell growth, migration, angiogenesis, proliferation, differentiation, apoptosis, and metabolism	[5,28,31,34,48]
PI3K/AKT/mTOR Pathway	Regulates cell proliferation, growth differentiation, and cell apoptosis inhibition	[5,28,31,34,37,48]
HRAS; KRAS	Proto-oncogenes, GTPase; regulates cell proliferation, migration, growth, survival, and differentiation	[1,5,31,36,37,40,49]; [1,5,28,31,36,37,40,49]
EGFR	Epidermal growth factor receptor; activates signaling pathways, regulates tissue regeneration, construction, homeostasis, cellular proliferation, apoptosis, and differentiation	[31,35,37,50]
FAT1	FAT atypical Cadherin 1; regulates mitochondrial respiration, cellular migration, and proliferation	[1,5,27,42]
PTEN	Phosphatase and tensin homolog; activates PI3K/AKT/mTOR pathway and facilitates cellular migration, growth, proliferation, signaling, protein synthesis, and DNA repair	[5,27,31,46]

**Table 2:** Additional genes cited in 2–3 primary sources as mutationally relevant and potential contributors to cSCC tumorigenesis. Description references [107,108,109,110,111,112,113,114,115,116,117,118,119,120,121].

Gene(s)	Description	References
CCND1	Cyclin D1, a regulator of CDK kinases; regulates cell cycle and metastasis	[5,31,37]
CREBBP	CREB-binding protein; regulates tumor response, cell growth, and division	[5,31,42]
EP300	E1A-binding protein P300; cell growth, proliferation, maturation, differentiation	[5,31,42]
ERBB4	Erb-b2 receptor tyrosine kinase 4; tumor suppressor, cellular response to EGFR	[31,37,42]
EZH2	Enhancer of zeste 2 polycomb repressive complex 2; silences tumor suppressor genes and regulates apoptosis, cell proliferation, and cycle regulation	[5,31,42]
FGFR3	Fibroblast growth factor receptor 3; regulates cell proliferation, survival, migration, and angiogenesis	[31,40,50]
KMT2C/KMT2D (MLL3)	Histone lysine methyltransferase 2C/2D; regulates transcriptional coactivation, genome maintenance, and histone methylation	[1,37,42]
TGFBR2	Transforming growth factor beta receptor 2; regulates cell differentiation, proliferation, maturation, and tissue homeostasis	[36,38,47]
AJUBA	Ajuba LIM protein; regulates epidermal cell differentiation and homeostasis	[1,5]
ARID2	AT-rich interaction domain 2; chromatin remodeling	[5,31]
ATM	Ataxia telangiectasia mutated; regulates stress response and DNA repair	[37,42]
BRAF	Serine/threonine protein kinase; regulates MAP/ERK pathway, cell division, differentiation, and secretion	[31,42]
BRCA2	Breast cancer gene 2; regulates genomic stability, DNA repair, and damage response	[37,39]
CASP8	Cysteine-aspartic acid protease; regulates cell survival and apoptosis	[1,5]
KNSTRN	Kinetochore-localized astrin/SPAG5-binding protein; regulates chromosomal stability and division	[103,121]
LRP1B	Low-density lipoprotein receptor-related protein 1B; regulates cell signaling and migration	[37,39]
NFE2L2	Nuclear factor erythroid 2-related transcription factor 2; regulates oxidative stress and inflammatory response	[1,5]
CARD11	Caspase recruitment domain family, member protein 11; regulates immune response through lymphocyte activation	[31,43]
MYC	Myelocytomatosis oncogene; regulates mRNA translation, stress response, immune response, cell cycle, proliferation, differentiation, apoptosis	[31,40]
RIPK4	Receptor-interacting serine/threonine kinase protein 4; regulates keratinocyte differentiation, cutaneous inflammation, and wound repair	[1,31]

**Table 3:** Phase II clinical trials conducted on cSCC or HNSCC with the respective overall response rates (ORRs). ORRs were calculated using the number of patients who showed partial or complete response, using the intent to treat population. * ORRs for the neoadjuvant trial are reported using major pathological responses (<10% tumor volume) and complete pathological responses.

Treatment(s)	Patient Population	ORR	References
Immunotherapy Monotherapy: cemiplimab	advanced metastatic cSCC	47% (*n* = 59)	[11]
Immunotherapy Monotherapy: pembrolizumab	recurrent or metastatic cSCC	34% (*n* = 105)	[174]
Immunotherapy Monotherapy: pembrolizumab	primary unresectable cSCC	42% (*n* = 57)	[83]
Neoadjuvant Immunotherapy: cemiplimab	advanced resectable cSCC	63% (*n* = 79) *	[12]
EGFR Monotherapy: cetuximab	primary unresectable cSCC	28% (*n* = 36)	[175]
EGFR Monotherapy: panitumumab	advanced, metastatic, or recurrent cSCC	31% (*n* = 16)	[176]
EGFR Monotherapy: erlotinib	recurrent or metastatic cSCC	8% (*n* = 39)	[177]
EGFR Monotherapy: gefitinib	recurrent and or metastatic cSCC	15% (*n* = 40)	[178]
Radiation + EGFR Inhibitors: cetuximab	locally advanced HNSCC	91% (*n* = 36)	[179]
Immunotherapy + EGFR Inhibitors: pembrolizumab and cetuximab	recurrent or metastatic HNSCC	45% (*n* = 33)	[180]
Immunotherapy + EGFR Inhibitors: nivolamb and cetuximab	previously treated recurrent or metastatic HNSCC; first-line recurrent or metastatic HNSCC	23% (*n* = 47); 33% (*n* = 48)	[181]
